# Surgical treatment of Boerhaave syndrome in the past, present and future: updated results of a specialised surgical unit

**DOI:** 10.1308/rcsann.2024.0020

**Published:** 2024-04-02

**Authors:** T Triantafyllou, P Lamb, R Skipworth, G Couper, C Deans

**Affiliations:** Department of Surgery, Royal Infirmary of Edinburgh, Scotland, NHS Lothian, UK

**Keywords:** Boerhaave syndrome, Spontaneous oesophageal perforation, Ruptured oesophagus, Sepsis

## Abstract

**Introduction:**

Boerhaave syndrome is a rare clinical entity associated with high rates of morbidity and mortality. Early recognition of the symptoms, and identification of the site and extension of the injury are key in improving the prognosis.

**Methods:**

This study presents data on the mortality, morbidity and length of hospital stay in patients diagnosed with Boerhaave syndrome. The data were retrieved from a prospectively collected database in a single surgical unit between 2012 and 2022. The study makes a comparison with the surgical outcomes of the previous decade.

**Results:**

Some 33 patients were diagnosed with Boerhaave syndrome and were treated surgically between 2012 and 2022 in a specialist upper gastrointestinal surgical unit. All patients underwent standard surgical repair (in-theatre diagnostic endoscopy, T-tube placement through thoracotomy and feeding jejunostomy through laparotomy). The mean size of the defects in the oesophageal lumen was 3.3cm. Delayed presentation was noted for 13 patients (39%); 8 patients (24%) died in hospital, and 19 patients (58%) developed postoperative complications. Mortality was similar to the rate recorded for the 20 patients from the previous decade (24% vs 20%, respectively). The mean length of hospital stay was 41 days, and was comparable to the 35.7 days reported between 1997 and 2011.

**Conclusions:**

Early and aggressive management of spontaneous oesophageal rupture ameliorates the postoperative recovery and prognosis. The surgical results of our unit were found comparable to the previous decade in the population of patients who were treated surgically.

## Introduction

Boerhaave syndrome, initially described in 1724 by Hermann Boerhaave, has been extensively investigated in the literature with numerous reports. Baron Jan von Wassenaer, a Dutch admiral, was one of the first cases ever reported. The admiral suffered acute left-sided chest pain after severe vomiting following a feast and did not survive the incident.^1^ Since then, the condition has been known as spontaneous rupture of the oesophagus. It comprises 15% of all oesophageal perforations and is considered a full-thickness injury that may prove fatal in 40% of patients. The incidence of the disease has been estimated to be about 3.1 per 1,000,000 individuals per year.^2^

Severe emesis and violent retching, mainly reported among middle-aged men with an extensive history of alcohol abuse, are usually the trigger for the injury. The left posterolateral aspect of the distal third of the oesophagus is the most common site of rupture. Clinical presentation can vary from completely asymptomatic to developing irreversible septic shock, depending on the level of the injury and the spillage in the mediastinum. Mackler's triad is the typical presentation consisting of vomiting, subcutaneous emphysema and chest pain; however, the severity of the clinical manifestation may vary.

A thorough history and imaging including computed tomography (CT) scanning and, in stable patients, a contrast swallow oesophagogram, usually reveal the defect in the oesophagus.^3,4^ Laboratory tests contribute to the differential diagnosis aiming to exclude pancreatitis or myocardial infarction and other urgent conditions. In the acute setting, a patient with sepsis may present with an oesophageal wall thickening and/or defect, subcutaneous or mediastinal emphysema or pleural effusions in the CT scan. In stable patients, a contrast oesophagogram with a water-soluble contrast agent may show extravasation in the mediastinum, and guides the surgeon in assessing the size of the defect, the height of the injury in the oesophageal body and the space where contamination is more prominent.

A transthoracic approach is recommended when there is significant pleural contamination. The time between the episodes of vomiting and the onset of symptoms is key in deciding the optimal therapeutic approach and estimations of prognosis. Surgical intervention includes primary oesophageal repair ideally within the first 24h after the rupture or placement of a T-tube in the defect. Different approaches such as thoracotomy, video-assisted thoracoscopy or three-dimensional thoracoscopy have been described in the literature. Delayed repair may also require debridement and decortication of the mediastinum. Oesophagogastric diversion may be required in damage-control conditions as a life-saving bridging intervention until the patient overcomes the septic shock. By contrast, conservative or endoscopic management is usually reserved for contained ruptures in stable patients with good results.^5^

## Methods

This study presents data on the management of patients diagnosed with Boerhaave syndrome. The data were retrieved from a prospectively collected database in a single surgical unit for a decade between 2012 and 2022, and are compared with surgical outcomes from the previous decade (1997–2011).^6^ Baseline demographic characteristics of the patients, detailed history, comorbidities and findings on admission (duration of symptoms before treatment, clinical findings, imaging) were reviewed. Morbidity, mortality and the length of hospital stay were the primary endpoints of the study.

Patients with iatrogenic oesophageal injuries and those who underwent conservative management with medical treatment were excluded. Confirmation of the location and extent of the injury was based on endoscopic findings or CT scanning upon presentation to the emergency department. After initial resuscitation, surgical repair of the oesophageal defect combined with wash-out of pleural or peritoneal effusions, drainage using thoracic tubes and nutritional support through feeding jejunostomy was the standard approach in all patients. Although early recognition of oesophageal injuries might be an indication for primary closure of the defect in selected patients, in our case series all perforations were closed with a T-tube in the oesophageal lumen because of a more delayed presentation.

The use of T-tubes through the perforated site of the oesophageal wall in patients with delayed presentation was initially described several decades ago and remains an option in selected cases, especially when endoscopic sealing techniques are not available. In our centre, the placement of a size 16 or 18 French T-tube with interrupted 3-0 PDS sutures through the defect during thoracotomy remains the preferred strategy. The insertion point is usually the fifth or sixth intercostal space in the midaxillary line with a short tract from the defect. Nutritional support using the feeding jejunostomy is initiated on the first postoperative day, whereas oral intake with liquid diet is initiated once there is no leak of contrast around the tube on an oral contrast study around day ten. The T-tube is usually removed in the outpatient clinic between six and eight weeks after insertion, providing there is no leak around the tube and the patient has been tolerating a soft oral diet. Supportive and targeted postoperative care with hydration, antibiotics, pain control and nutritional support was provided in the intensive care unit or surgical ward. Prolonged hospital stay or repeat CT scan or contrast oesophagogram with oral contrast was deemed necessary for patients with ongoing sepsis and suspected persistent leak.

## Results

### Clinical and radiological evaluation

A total of 33 patients (10 female and 23 male) treated for Boerhaave syndrome between 2012 and 2022 were identified in our prospectively collected database. Mean patient age was 65 years (range 40–89 years). Delayed presentation to the hospital since the onset of symptoms (more than 24h) was reported in 13 patients (40%). Most patients presented with a history of vigorous vomiting (17 patients), six complained of chest pain, four had Meckler's triad (vomiting, emphysema, chest pain) and two presented with fever (Table 1). Further information with regards to the symptoms was not identified for the remaining patients (*n* = 4). Eleven patients were referrals from other hospitals. Bilateral pleural effusions were the predominant radiological finding during the diagnostic workup in the vast majority (23 patients) of the CT scans performed.

**Table 1:** Presenting symptoms (*n* = 29 of 30)

Presenting symptom	No. of patients
Vigorous vomiting	17
Chest pain	6
Typical Meckler's triad	4
Fever	2

### Treatment pathway

After initial resuscitation with an oxygen supply when required, broad-spectrum antibiotics, intravenous fluid replacement and pain control, aggressive treatment with in-theatre flexible endoscopy (performed in 29 of 33 cases) and surgical repair were attempted. The distal oesophagus was the site of injury in all cases and only two injuries extended to the middle oesophagus. The mean size of the defects was 3.3cm (range 1–8cm) based on the intraoperative endoscopic or macroscopic findings.

**Figure 1:**
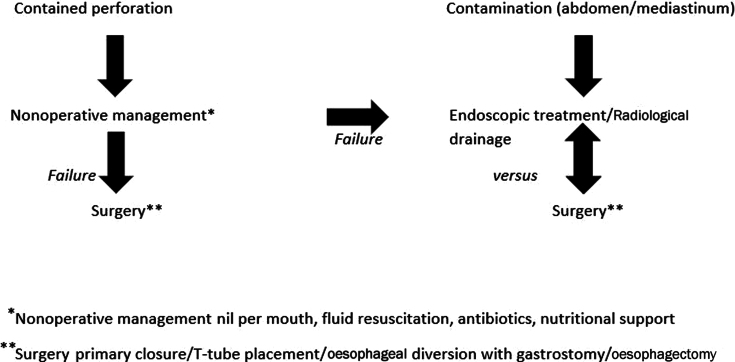
Available treatment pathways in oesophageal perforation

Given that most patients presented with bilateral involvement of the mediastinum, the standard approach in all cases included in this study consisted of urgent thoracotomy and placement of a T-tube in the oesophageal lumen through the defect, wash-out of the mediastinum and drainage using chest tubes to achieve a controlled fistula in persistent leak. The procedure was completed with a laparotomy for placement of a feeding jejunostomy tube. Figure 1 presents an algorithm for the different options available.

### Mortality rate

Eight patients (24%) died in hospital (Table 2). Among them, three died within the first postoperative week as a result of delayed presentation and irreversible multiorgan failure. The other five deaths resulted from renal failure (*n* = 1), pneumonia and respiratory failure (*n* = 4). The mean age of these eight patients was 65 years and the time from the onset of their symptoms to the definitive surgical treatment was more than 48h for four patients. Overall, 13 of 33 patients presented to the emergency department more than 48h after the onset of their symptoms. The in-hospital mortality of patients with delayed presentation was 31%.

**Table 2:** Mortality (24%) and aetiology of deaths (*n* = 8)

Patient	Time from symptoms to surgery (h)	Cause of death	Postoperative day of death
1	48	Multiorgan failure	3
2	24	Multiorgan failure	7
3	24	Pneumonia–respiratory failure	13
4	48	Pneumonia–multiorgan failure	16
5	48	Pneumonia–respiratory failure	12
6	72	Multiorgan failure	11
7	24	Renal failure	86
8	24	Multiorgan failure	6

### Surgical outcomes and morbidity

Nineteen patients (57%) presented postoperative short- or long-term complications (Table 3). Three patients developed a surgical site infection at the insertion site of their chest tubes or their thoracotomies and were treated with antibiotics, whereas two underwent surgical wash-out of subcutaneous collections. No other reoperations were recorded. Eight patients recovered from respiratory complications after their surgical repair (three with bronchopneumonia treated with antibiotics, four with recurrent pleural effusions drained under interventional radiology and one underwent tracheostomy for respiratory failure). One patient with a background history of renal transplantation was treated with intravenous antifungal therapy (positive blood cultures) and was resuscitated from acute kidney injury. Another patient developed postoperative bleeding presented as melaena; however, no intervention was required and the patient was treated conservatively with a blood transfusion. One patient underwent a repeat CT scan for investigation of acutely deranged liver function enzymes and was diagnosed with thrombosis of the left hepatic artery. Follow-up with repeat imaging and antithrombotic therapy were recommended.

**Table 3:** Morbidity (58%) after resuscitation and interventional management

	Complication	No. of patients (*n* = 19 of 33)	Treatment
Short-term postoperative complications	SSI	*n* = 3	Antibiotics Surgical drainage (2 of 3)
	Respiratory complications	*n* = 8 (bronchopneumonia, *n* = 3; pleural effusions, *n* = 4; respiratory failure, *n* = 1)	Antibiotics Drainage (IR) Tracheostomy
	Fungal systemic infection	*n* = 1	Intravenous antifungals
	Coagulopathies	*n* = 2 (GI bleeding, *n* = 1; arterial thrombosis, *n* = 1)	Transfusion Anticoagulation
Long-term postoperative complications	Oesophageal stenosis	*n* = 5	Endoscopic dilatation

GI = gastrointestinal; IR = interventional radiology; SSI = surgical site infection

The mean length of hospital stay for the patients who recovered postoperatively and were discharged from the hospital was 41 days. Five patients represented with symptoms secondary to oesophageal strictures postdischarge and underwent endoscopy and balloon dilatation.

Compared with the management of patients treated for Boerhaave syndrome between 1997 and 2011 in our unit, data on all consecutive patients diagnosed and treated for the same condition during the past decade resulted in a few interesting observations. Selecting a common strategy using the T-tubing technique in recent years is the basic difference from the past and can be attributed to the size of the defects in the oesophageal wall, as well as the delayed presentation in 40% of these patients making primary closure a less-favourable option. Although the mean length of hospital stay and overall mortality were similar between the two periods analysed, morbidity was higher in the current analysis. Older age (mean 65 years compared with 57 years in the previous study) and the challenging size and location of the perforations recorded between 2012 and 2022, leading to surgical rather than conservative management, could explain the differences in the complication rates (Table 4). Long-term complications that were treated conservatively, mainly consisting of dysphagia because of oesophageal stenosis and recurrent local infection, were identified in six patients during 1997–2011 and in five patients during 2012–2022.

**Table 4:** Comparison of patient characteristics, treatment pathways and results between the two periods (1997–2011, 2012–2022)

	1997–2011 (*n* = 20)	2012–2022 (*n* = 33)
Mean age, years	57 (range: 20–83)	65 (range: 40–89)
Sex	10 female/10 male	10 female/23 male
Treatment modality	Nonoperative (*n* = 3) Primary repair (*n* = 5) T-tube (*n* = 11) Irreparable (*n* = 1)	T-tube placement (*n* = 33)
Postoperative morbidity	50% (*n* = 10)	57% (*n* = 19)
Mortality	20% (*n* = 4) (T-tube, *n* = 2; primary repair, *n* = 1; irreparable, *n* = 1)	24% (*n* = 8)
Mean length of hospital stay	35.7 days after T-tube 20.5 days after primary closure	41 days

## Discussion

The prognosis of Boerhaave syndrome largely depends on the duration of symptoms and the early recognition and management of the oesophageal injury. The Pittsburgh Severity Score summarises these parameters, stratifies the severity of the clinical presentation for each patient and may guide an individualised therapeutic pathway.^7^ Age, heart rate, level of leukocytes, presence of pleural effusions, high temperature, hypotension, non-contained leak, time to final diagnosis, respiratory impairment and any type of cancer diagnosis are all taken into consideration and assessed on a scale of 1–3 with a maximum total score of 18. The Pittsburgh Severity Score has proven significantly predictive of the complications after spontaneous oesophageal perforation and its use has been supported in the literature as an accurate scoring tool in the initial evaluation of these patients.^7^

Treatment is tailored to the patient's presentation and general condition, comorbidities, size and location of the defect, time to diagnosis and degree of tissue inflammation.^8^ The available therapeutic options are the conservative approach, endoscopic interventions and surgery, although fluid replacement, broad-spectrum antibiotics and antifungals, and aggressive supportive care by the anaesthetic team and the intensive care unit are crucial.^9^

Surgical repair of the defect and drainage of the mediastinum have long been considered the gold standard for early perforations, and previous studies have shown good results.^10^^–12^ Irrigation, debridement and drainage of the thoracic cavity are also significant steps to avoid established mediastinitis. Approximation of the defect with interrupted stitches and creation of an omental patch may be an option. However, primary suturing is not always technically feasible because of the level or extent of the injury or the prolonged time to diagnosis, and oesophageal diversion with cervical oesophagostomy with a second-stage reconstruction may be inevitable.^13^ Minimally invasive techniques in specialised units (video assisted thoracoscopic surgery (VATS), thoracoscopy) can also be considered for haemodynamically stable patients as long as exposure of the injury can be satisfactory and manoeuvres allow for a safe repair.^14,15^ Moreover, placement of a feeding jejunostomy allows for immediate enteral nutrition, although some authors have also reported the use of venting gastrostomy for safe decompression of the stomach in the treatment of spontaneous or iatrogenic oesophageal perforation in selected patients.^16^ Intraoperative endoscopic management can also be combined with debridement and drainage of the mediastinum through VATS.^17^ Oesophageal stenting, application of endo-clips and the use of endoluminal vacuum therapy (EVAC) have all been described previously with promising results.^18^^–20^ The time interval between the intervention and reassessment with imaging (oesophagogram or CT scanning) allows for sealing of the gastrointestinal (GI) tract and recovery from the rupture. Small defects may be amenable to clips, whereas high-risk patients who are poor surgical candidates may benefit from a less-invasive approach such as intraluminal stenting.

EVAC connected to a sponge may be another option depending on the findings and the extent of contamination.^21^ This technique has been proposed to control oesophagopleural fistulas; however, evidence about the results is still scarce and only a few cases have been reported in the literature. Interestingly, a combination of stenting and vacuum-assisted closure technology (VACStent) in selected patients with oesophageal leak has the potential to prevent stent migration and improve healing with the application of negative pressure.^18^ Furthermore, EVAC may be an alternative option following a failure-to-rescue primary repair.^20^

In this study, the morbidity and mortality rates were comparable to the case series reported in our previous analysis of cases diagnosed and treated between 1997 and 2011 in the same unit. Both morbidity and mortality remain high despite advances in the diagnostic tools and the variety in treatment options. The findings may reflect the delayed presentation of these patients to a specialist centre because of either neglected symptoms or difficulties in the differential diagnosis when referrals are made from other hospitals. This may be attributed to the fact that the Boerhaave syndrome can mimic several other pathological conditions. Hence, valuable time during thorough workup may be lost until final diagnosis is established. Therefore, high suspicion mainly based on the patient’s targeted history can be time-saving and expedite the time to source control and definitive treatment.

## Conclusion

Awareness and knowledge of spontaneous rupture of the oesophagus have changed dramatically over the past years as a result of more cases being reported and treated, as well as centralisation of specialised units in upper GI surgery. Advanced endoscopy and interventional radiology have led to promising results providing more favourable outcomes in selected cases with Boerhaave syndrome. Nevertheless, surgical treatment remains the cornerstone in patients with gross contamination following oesophageal rupture. The results for patients diagnosed and treated for Boerhaave syndrome in our unit were comparable with the past decade; however, endoscopic modalities such as stenting or EVT may be valid alternatives for future consideration. Individualisation with a step-up approach when necessary under the care of specialised physicians is the state of the art in the modern management of this challenging syndrome.

### Learning points

• Early diagnosis of Boerhaave syndrome can improve the outcomes of treatment.
• A contrast swallow oesophagogram or a CT scan with oral contrast are the most common diagnostic tools.
• Treatment options are tailored based on the patient’s condition, background history and the type of the oesophageal injury. The length of time between the rupture and patient presentation is also crucial in the prognosis.
• Primary suturing for early-presented ruptures or creation of a controlled fistula or closure with a T-tube in the oesophageal lumen can be attempted through a thoracotomy or thoracoscopy. An abdominal approach is rarely indicated.
• Endoscopic interventions combined with interventional radiology in cases with effusions or abscess cavities and/or empyema may be indicated in selected patients.
